# Assessment and agreement of the CT appearance pattern and its severity grading of radiation-induced lung injury after stereotactic body radiotherapy for lung cancer

**DOI:** 10.1371/journal.pone.0204734

**Published:** 2018-10-04

**Authors:** Takaya Yamamoto, Noriyuki Kadoya, Yohei Morishita, Yoshinao Sato, Haruo Matsushita, Rei Umezawa, Yojiro Ishikawa, Noriyoshi Takahashi, Yu Katagiri, Ken Takeda, Keiichi Jingu

**Affiliations:** 1 Department of Radiation Oncology, Tohoku University Graduate School of Medicine, Sendai, Japan; 2 Department of Diagnostic Radiology, Tohoku University Graduate School of Medicine, Sendai, Japan; University of South Alabama Mitchell Cancer Institute, UNITED STATES

## Abstract

**Purpose:**

Radiographic severity of radiation-induced lung injury (RILI) has not been well-studied. The goal of this study was to assess the CT appearance pattern and severity of RILI without consideration of the clinical presentation.

**Material and methods:**

A total of 49 patients, 41 with primary lung cancer and 8 with metastatic lung cancer, were treated by 4-fraction stereotactic body radiotherapy (SBRT). RILI after SBRT was separately assessed by two observers. The early and late CT appearance patterns and CT-based severity grading were explored.

**Results:**

The median follow-up period was 39.0 months. In the early CT findings of observers 1 and 2, there was diffuse consolidation in 15 and 8, diffuse ground glass opacity (GGO) in 0 and 0, patchy consolidation and GGO in 17 and 20, patchy GGO in 3 and 3, and no changes in 10 and 14, respectively (kappa = 0.61). In late CT findings of observer 1 and 2, there were modified conventional pattern in 28 and 24, mass-like pattern in 8 and 11, scar-like pattern in 12 and 12, and no changes in 1 and 2, respectively (kappa = 0.63). In the results of the CT-based grading by observers 1 and 2, there were grade 0 in 1 and 2, grade 1 in 10 and 14, grade 2 in 31 and 29, grade 3 in 7 and 4, and none of grade 4 or more, respectively (kappa = 0.66). According to multivariate analyses (MVA), the significant predicting factors of grade 2 or more CT-based RILI were age (p = 0.01), oxygen dependence (p = 0.03) and interstitial shadow (p = 0.03).

**Conclusions:**

The agreement of the CT appearance and CT-based grading between two observers was good. These indicators may be able to provide us with more objective information and a better understanding of RILI.

## Introduction

Stereotactic body radiotherapy (SBRT) for lung cancer is performed worldwide [1–3]. Radiation-induced lung injury (RILI) is a primary complication after SBRT and consists of acute lung injury (i.e., radiation pneumonitis) and chronic lung injury (i.e., radiation fibrosis) [4–5]. In recent years, an increasing number of reports have been published regarding RILI. Some have reported on the early and late CT appearance patterns after SBRT [6–9]. Others have analyzed the clinical and radiotherapeutic parameters regarding RILI, especially focusing on the development of symptomatic RILI [10–12].

In most cases, RILI is graded according to the Common Terminology Criteria for Adverse Events (CTCAE). Although CTCAE grading of pulmonary fibrosis includes radiographic pulmonary fibrosis, it is not appropriate for RILI after SBRT considering the CT appearance pattern after SBRT. CTCAE grading of pneumonitis deals with the symptomatic and therapeutic factors for RILI, but cannot assess the radiographic severity. It is true that symptomatic RILI is important, and many reports have emphasized the predicting factors of symptomatic RILI. However, the comorbidities of the patient, baseline respiratory function, subclinical interstitial lung disease, performance status or subjectivity of physicians can affect symptomatic complaints [10–15]. Thus, the radiological appearance of RILI is not always accompanied by clinical symptoms [16]. For example, many doctors have difficulty assessing RILI of oxygen-dependent patients treated with home oxygen therapy (HOT) because they frequently develop dyspnea and require an increase of oxygen flow in spite of a moderate radiographic change. These symptoms can be caused not only by RILI but also by progression of their underlying disease and poor pulmonary preservation. In this case, doctors would decide on the best fit category to grade RILI according to CTCAE, but the agreement between doctors would be low. This study aimed to objectively assess RILI and gather information that can be masked by clinical findings. In this study, one radiologist and one radiation oncologist separately assessed early and late CT appearances and graded late RILI using a CT-based severity grading scale after SBRT. In addition, the predictive factors of CT-based RILI grading were explored.

## Materials and methods

### Patients and treatments

This retrospective study was approved by the Ethical Committee of Tohoku University Hospital (reference number: 2016–768), and informed consent was obtained from all patients. SBRT for a non-centrally located lung tumor was assigned to a 4-fraction schedule at our institute. Patients who were treated with 4-fraction SBRT and for whom 6 months or more of follow-up CT data were available were included in this study. A total of 49 eligible patients were treated between December 2007 and August 2015. There were 41 patients with primary lung cancer and 8 with metastatic lung cancer. Six patients had received HOT treatment at the time of receiving SBRT (HOT patients), and all of these patients were diagnosed with chronic obstructive pulmonary disease. The characteristics of the patients and tumors are shown in Table 1.

**Table 1:** Baseline patient demographic characteristics.

Characteristics	Number (%)
Patients	49
Age, median	77 (range: 48–88)
Sex	
-Male	35 (71%)
-Female	14 (29%)
Pack-years smoked, median	29 (range: 0–100)
-Male, pack-years, median	40 (range: 0–100)
-Female, pack-years, median	0 (range: 0–30)
ECOG PS	
-PS 0–1	42 (86%)
-PS 2–3	7 (14%)
Oxygen-dependent (HOT)	6 (12%)
-Oxygen flow range, L/min	1.0–3.0
-Male/Female	6/0
Interstitial shadow	
-Yes	3 (6%)
-No	46 (94%)
Operability	
-Yes	16 (33%)
-No	33 (67%)
Tumor diameter, median, mm	19 (range: 10–39)
Tumor location	
-Upper lobe	27 (55%)
-Other lobes	22 (45%)
Pathology	
-Adenocarcinoma	11 (22%)
-Squamous cell carcinoma	8 (16%)
-Clinically diagnosed primary cancer	22 (45%)
-Clinically diagnosed metastatic cancer	8 (16%)
PTV dimension, median, cc	39.6 (range: 9.7–112.7)

Abbreviations: ECOG, Eastern Cooperative Oncology Group; HOT, home oxygen therapy; PTV, planning target volume.

In SBRT, a vacuum pillow (Vac-loc, Med-tek, Orange City, IA) was used to immobilize each patient. An X-ray simulator (Ximatron or Acuity, Varian Medical Systems, Palo Alto, CA), 4-D CT, or both were used to evaluate intrafractional lung tumor motion. If the respiratory amplitude was larger than 10 mm, the abdominal compression or breath hold method was used to reduce the internal target volume (ITV) margin. Planning CT scans were performed at intervals of 2.5 mm (GE Light Speed Qxi, GE Healthcare, Waukesha, WI). Gross tumor volume (GTV) was defined as the visible extent of the tumor on planning CT images. ITV was typically created by 10 respiratory phases generated from 4-D CT. For ITV, a 0–5 mm margin was added to account for microscopic extension and then was expanded by 5 mm in all directions a to account for the uncertainty of the set up and to form the planning target volume (PTV). Radiotherapy planning was performed using a 3-D radiotherapy planning system (Eclipse, Varian Medical Systems, Palo Alto, CA). SBRT was delivered using multiple coplanar and non-coplanar static beams with a linear accelerator (Clinac 23EX, Varian Medical Systems, Palo Alto, CA). Thirty-seven patients were prescribed 40 Gy in 4 fractions covering 95% of the PTV (D95), and 12 patients were prescribed 48 Gy in 4 fractions to the isocenter. The prescribed doses were delivered using 6 MV photons and 600 monitor units per minute.

### Early and late CT appearance patterns and CT-based grading scale of RILI

The CT appearance pattern was judged according to Linda’s classification of the CT findings [17]. Early appearance was defined as CT findings in the first 6 months after SBRT; late appearance was defined as CT findings after the first 6 months after SBRT. The early CT appearance pattern consisted of a diffuse consolidation, diffuse ground-glass opacity (GGO), patchy consolidation and GGO, patchy GGO, and no changes. The late CT appearance pattern consisted of a modified conventional pattern, mass-like pattern, scar-like pattern, and no changes.

For the CT-based severity grading scale, the modified RTOG/EORTC Late Radiation Morbidity Scoring Schema of the lung was used. The classification of the CT-based grading was as follows: grade 0, none; grade 1, slight radiographic appearance; grade 2, patchy radiographic appearance; grade 3, diffuse radiographic change <25% of the lung volume; grade 4; diffuse radiographic change ≥25% of the lung volume; and grade 5, death (Table 2). Because the late radiation scoring schema was used, follow-up CT 6 months after SBRT was used for the judgments. or grading, diffuse radiographic changes were used instead of dense radiographic changes of the RTOG/EORTC criteria because dense radiographic changes or similar changes (such as mass-like shadow) were sometimes observed after SBRT. Thus, the CTCAE of pulmonary fibrosis was referenced to define grades 3 and 4.

**Table 2:** CT-based radiological appearance according to the grading scale of RILI.

Grade	CT appearance
0	None
1	Slight radiographic appearances
2	Patchy radiographic appearances
3	Diffuse radiographic changes <25% of lung volume
4	Diffuse radiographic changes >25% of lung volume
5	(Death)

### Statistical analysis

The early/late CT appearance patterns and CT-based grading of RILI were assessed by two observers: one radiation oncologist and one radiologist (T.Y. and Y.M., with 9 and 5 years of experience, respectively). Each assessment was blinded, but the clinical and treatment information were open. The agreement between interobservers was measured using the kappa static [18]. Cohen's unweighted kappa was applied for the agreement of the CT appearance pattern, and the quadratic-weighted kappa was applied for the agreement of the grading. Interobserver agreement was categorized by kappa values, as follows: poor, <0.20; fair, 0.20–0.39; moderate, 0.40–0.59; good, 0.60–0.79; or excellent, >0.80. To perform radiotherapeutic parameter analysis, all treatment plans were recalculated with Acuros XB, version 11031. The parameter of V_*n* Gy_ was defined as the percentage volume of the lung that received *n* Gy or more. The time to an event was calculated from the first day of SBRT to the day an event was confirmed. The Cox proportional hazards model was used to perform univariate analyses (UVA) and multivariate analyses (MVA). A stepwise backward elimination/forward addition approach using the Akaike information criterion (AIC) was applied to build the best MVA model. A p value less than 0.05 was defined as significant. EZR, version 1.35 (Saitama Medical Center, Jichi Medical University, Saitama, Japan), a modified version of R commander (R Foundation for Statistical Computing, Vienna, Austria), was used for Cox regression analyses [19].

## Results

### Treatment results

The median follow-up period was 39.0 months for all patients and 42.3 months for living patients. During follow-up, 14 patients died: 10 died from primary disease; 2 from another cancer; 1 from cerebral infarction; and 1 in an accident. No treatment-related deaths occurred. The median recalculated dose of D95 was 39.7 Gy (range, 34.3–43.4 Gy), and local tumor failure occurred in 9 patients during follow-up. Symptomatic RILI occurred in 10 patients, and steroids were administered to 4 patients. According to CTCAE, grade 0 was assessed in 10 patients; grade 1 in 29 patients; grade 2 in 9 patients; and grade 3 RILI in 1 patient. There were rib fractures in 7 patients.

### Assessment of RILI

**Fig 1:**
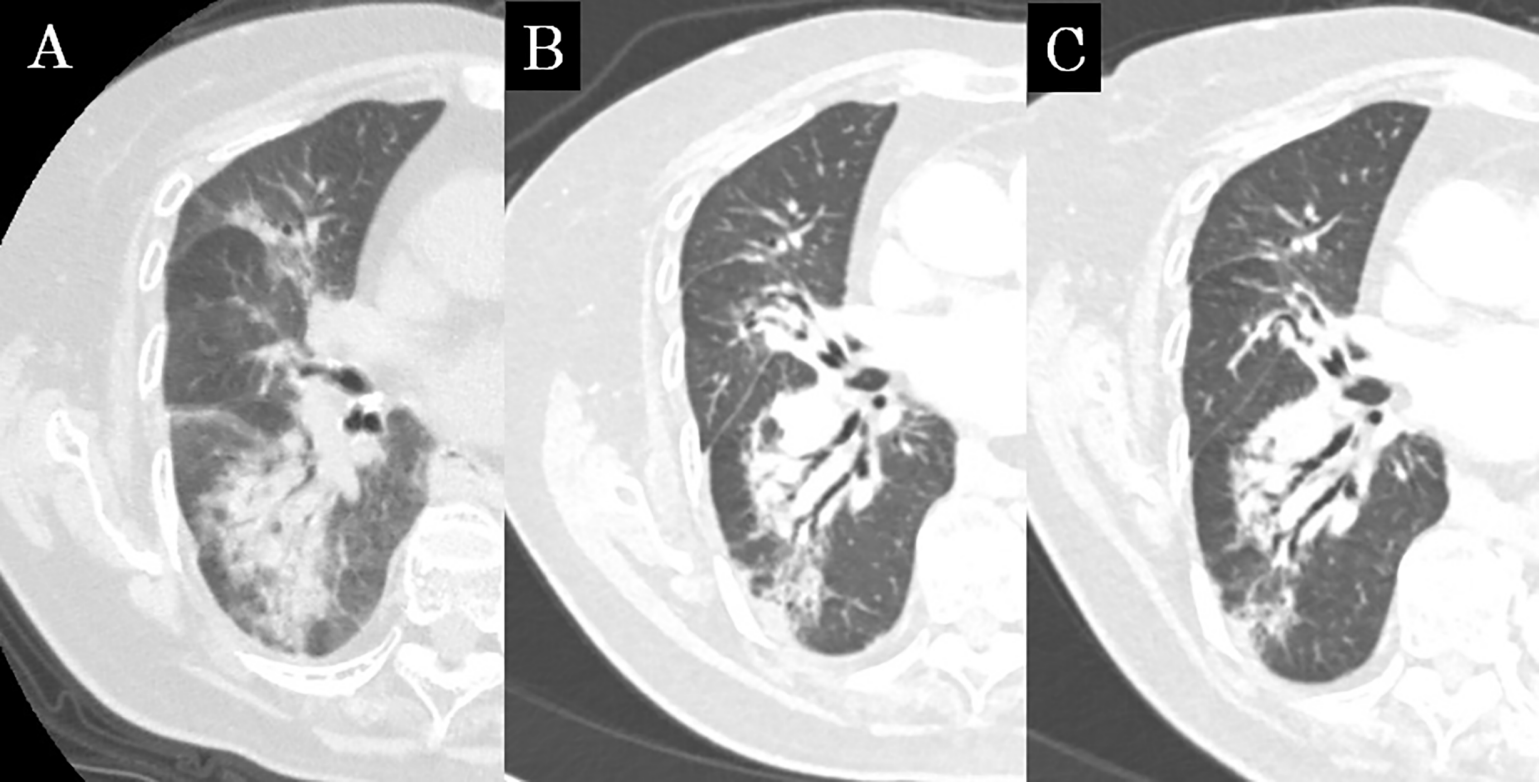
Concordance CT appearance, case 1. (a) The shadow extends to the right middle and lower lobes beyond the high-dose region; this was diagnosed as diffuse consolidation at 6 months after SBRT. (b) The shadow shows consolidation, volume loss and bronchiectasis; this was diagnosed as the modified conventional pattern at 21 months after SBRT. (c) The shadow remained at 45 months after SBRT.

**Fig 2:**
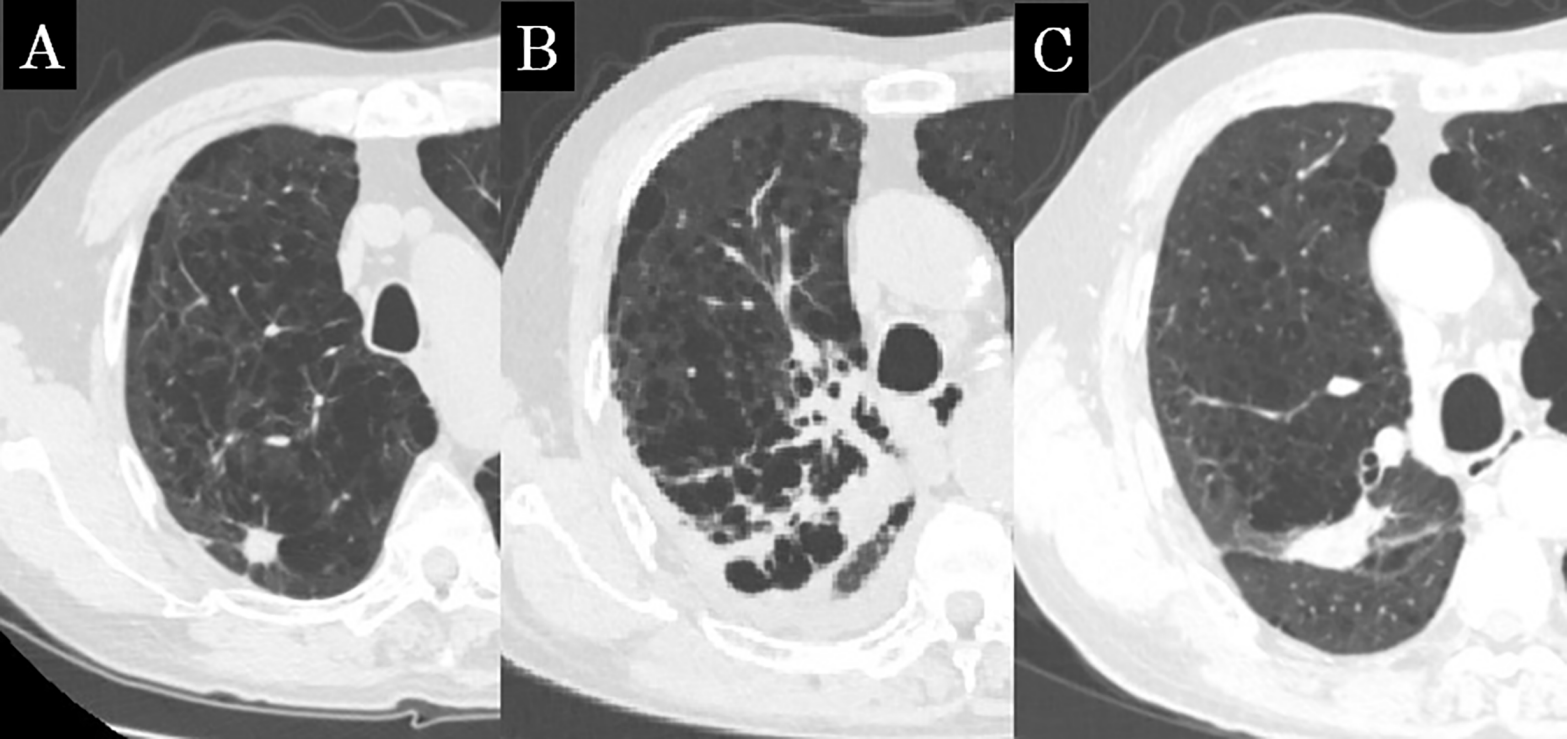
Concordance CT appearance, case 2. (a) Before SBRT, the lung tumor was located in the right upper lobe. (b) The consolidation expands beyond the high-dose region; this shadow was diagnosed as diffuse consolidation at 5 months after SBRT. (c) The shadow shrank, and only a linear opacity remained. This shadow was diagnosed as a scar-like pattern at 20 months after SBRT.

**Fig 3:**
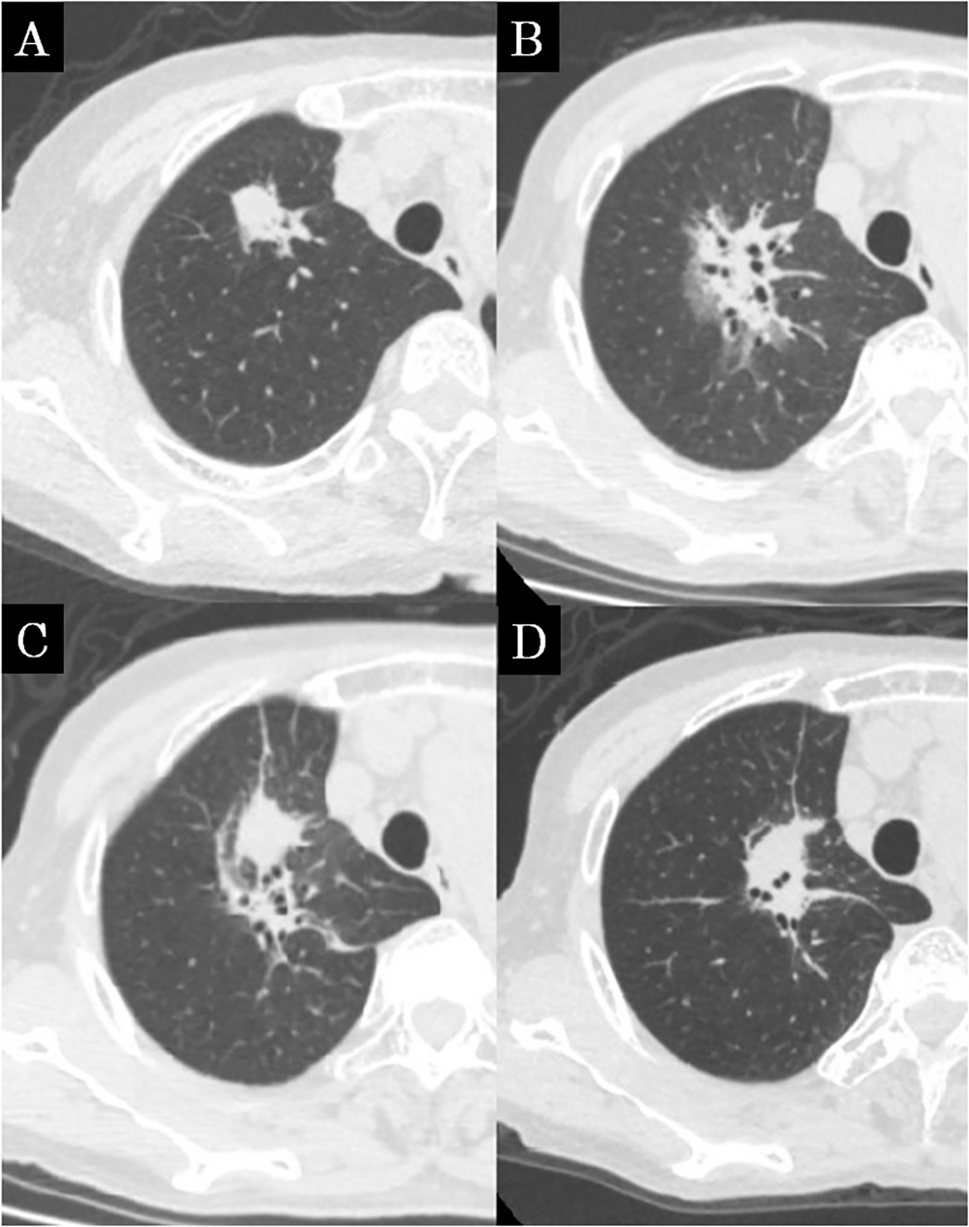
Concordance CT appearance, case 3. (a) Before SBRT, the lung tumor was located in the right upper lobe. (b) Patchy areas of hazy and consolidation are seen; this shadow was diagnosed as patchy consolidation and GGO at 5 months after SBRT. (c) The shadow forms a focal consolidation; this was diagnosed as a mass-like pattern at 17 months after SBRT. (d) The shadow shrank, but a mass-like shadow remained at 35 months after SBRT.

**Fig 4:**
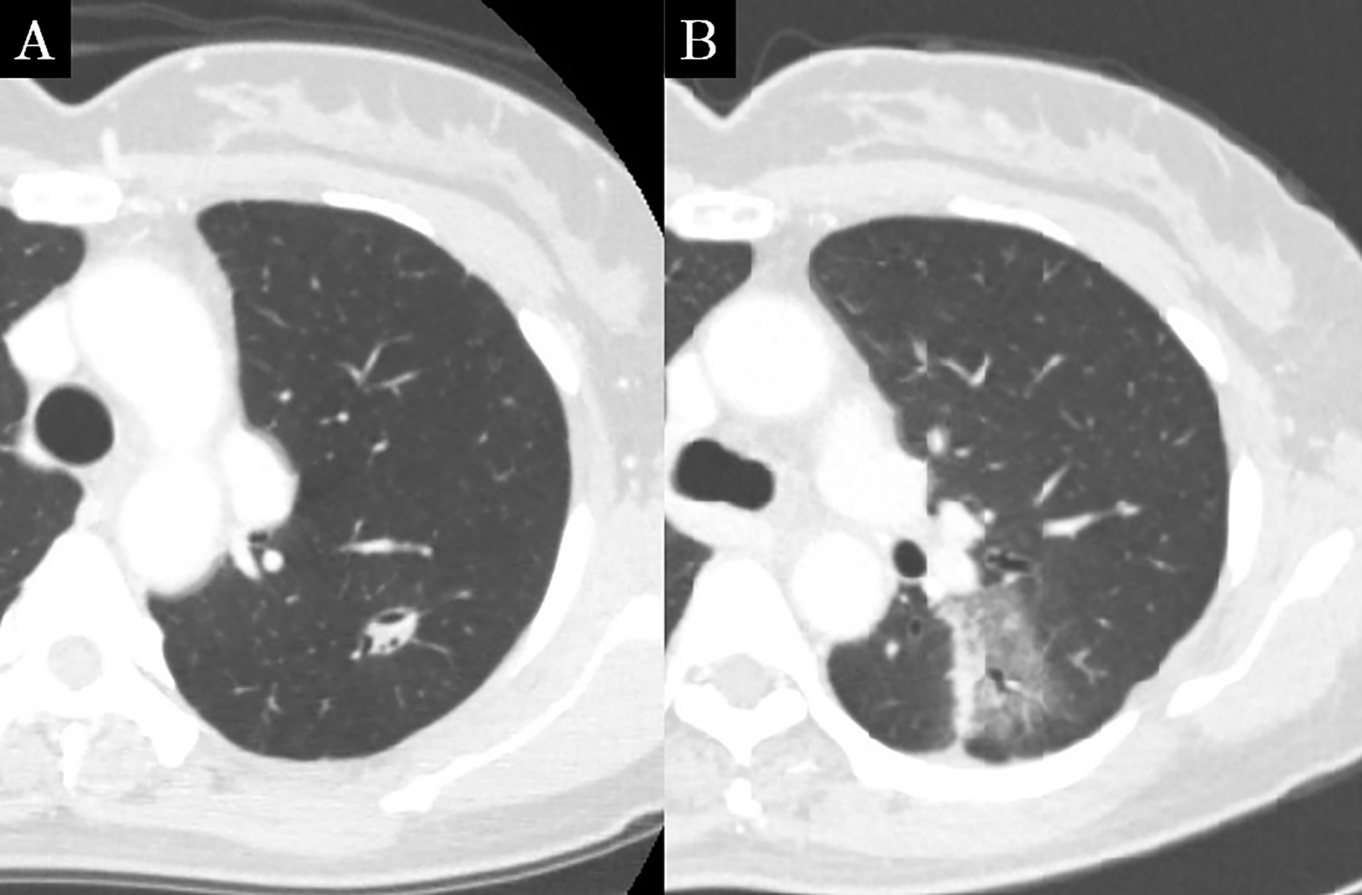
Concordance CT appearance, case 4. (a) Before SBRT, the lung tumor was located in the left lower lobe. (b) Patchy areas of hazy are seen; this shadow was diagnosed as patchy GGO at 3 months after SBRT.

The early and late CT findings judged by observer 1 and observer 2 are shown in Tables 3 and 4, and representative concordance CT images are shown in Figs 1–4. The mean time from the end of SBRT to diagnoses of early and late CT by observer 1 were 4.4 months (95% confidence interval [CI]: 3.9–4.9) and 22.2 months (95% CI: 18.5–25.9), respectively; those by observer 2 were 4.8 months (95% CI: 18.5–25.9) and 16.7 months (95% CI: 13.6–19.9), respectively. In the early CT findings of observer 1, there was diffuse consolidation in 15 patients (33.3%), diffuse GGO in no patients (0%), patchy consolidation and GGO in 17 patients (37.7%), patchy GGO in 3 patients (6.6%) and no changes in 10 patients (22.2%). In the late CT findings of observer 1, there were modified conventional patterns in 28 patients (57.1%), mass-like patterns in 8 patients (16.3%), scar-like patterns in 12 patients (24.4%) and no changes in 1 patient (2.0%).

**Table 3:** Early CT appearance pattern as judged by the two observers.

Observer 1	Diffuse consolidation	Diffuse GGO	Patchy consolidation and GGO	Patchy GGO	No changes	Total
Observer 2						
Diffuse consolidation	8	0	0	0	0	8
Diffuse GGO	0	0	0	0	0	0
Patchy consolidation and GGO	5	0	14	0	1	20
Patchy GGO	1	0	0	2	0	3
No changes	1	0	3	1	9	14
Total	15	0	17	3	10	45

Abbreviations: GGO, ground-glass opacity.

**Table 4:** Late CT appearance pattern as judged by the two observers.

Observer 1	Modified conventional pattern	Mass-like pattern	Scar-like pattern	No changes	Total
Observer 2					
Modified conventional pattern	22	0	2	0	24
Mass-like pattern	2	7	2	0	11
Scar-like pattern	4	0	8	0	12
No changes	0	1	0	1	2
Total	28	8	12	1	49

The CT-based late radiographic grading of RILI was assessed by observer 1 as follows: grade 0 in 1 patient (2.0%%), grade 1 in 10 patients (20.4%), grade 2 in 31 patients (63.2%), grade 3 in 7 patients (14.2%) and grade 4 or more in no patients. The CT-based late radiographic grading of RILI by observer 2 was assessed as follows: grade 0 in 2 patients (4.0%), grade 1 in 14 patients (28.5%), grade 2 in 29 patients (59.1%), grade 3 in 4 patients (8.1%) and grade 4 or more in no patients (Table 5).

**Table 5:** CT-based late radiographic grading of RILI as judged by the two observers.

Observer 1	Grade 0	Grade 1	Grade 2	Grade 3	Total
Observer 2					
Grade 0	1	1	0	0	2
Grade 1	0	8	6	0	14
Grade 2	0	1	23	5	29
Grade 3	0	0	2	2	4
Total	1	10	31	7	49

Abbreviations: RILI, radiation-induced lung injury.

### Interobserver variability

The unweighted kappa for the early CT appearance of RILI was 0.61 (95% CI: 0.43–0.80), which suggested that the agreement between interobservers was good. The unweighted kappa for the late CT appearance was 0.63 (95% CI: 0.45–0.82), which similarly indicated that the agreement between interobservers was good. The agreement for the CT-based late radiographic grading of RILI was also good (quadratic weighted kappa = 0.66; 95% CI: 0.23–1.00). However, the agreement between the CTCAE grade and radiographic grading by observer 2 was moderate (quadratic weighted kappa = 0.43; 95% CI: 0.20–0.67), and the agreement between the CTCAE grade and radiographic grading by observer 1 was not calculated because the observed concordance was smaller than the mean chance concordance.

### Cox regression analyses for CT-based RILI grades

Cox regression analyses for grade 2 or more RILI as assessed by observer 1 using only CT findings were performed. The results of UVA and MVA are shown in Table 6 and 7. There was a significant relationship between patients treated with HOT (hazard ratio [HR]: 0.21; 95% CI: 0.05–0.92; p = 0.03), the presence of an interstitial shadow (HR: 3.50; 95% CI: 1.01–12.0; p = 0.04), and a recalculated dose of D95 (per 1 Gy increase; HR: 1.20; 95% CI: 1.02–1.42; p = 0.02). In MVA, age (per 1 year old; HR: 0.95; 95% CI: 0.91–0.99; p = 0.01), patients treated with HOT (HR: 0.20; 95% CI: 0.04–0.88; p = 0.03) and the presence of interstitial shadow (HR: 4.06; 95% CI: 1.13–14.5; p = 0.03) emerged as significant factors.

**Table 6:** Univariate analyses (Cox regression) for grade 2 or more (the grading was based on the radiographic grading of RILI assessed by observer 1).

Variables	HR (95% CI)	P value
Age (per 1 year old)	0.98 (0.95–1.01)	0.37
Sex (Female vs Male)	1.31 (0.66–2.61)	0.43
PS (>2 vs ≤1)	0.88 (0.34–2.29)	0.80
HOT (Yes vs No)	0.21 (0.05–0.92)	0.03*
Pack-year smoking (per 1 pack-year)	0.99(0.98–1.00)	0.32
Operability (Yes vs No)	0.82 (0.40–1.68)	0.59
Interstitial shadow (Yes vs No)	3.50 (1.01–12.0)	0.04*
Treated lobe (Upper vs others)	0.56 (0.29–1.08)	0.08
Tumor diameter (per 1 mm)	1.02 (0.97–1.07)	0.32
PTV size (per 1 cc)	1.00 (0.98–1.01)	0.82
Dose of D95 (per 1 Gy)	1.20 (1.02–1.42)	0.02*
Lung V_5 Gy_ (per 1%)	1.00 (0.97–1.04)	0.60
Lung V_20 Gy_ (per 1%)	1.01 (0.93–1.08)	0.79
Overall treatment period (per 1 day)	0.93 (0.78–1.10	0.41

Abbreviations: RILI, radiation-induced lung injury; PS, performance status; HOT, home oxygen therapy; PTV, planning target volume; D95, covering 95% of the PTV.

*Statistically significant (p < 0.05).

**Table 7:** Multivariate analyses (Cox regression) for grade 2 or more (the grading was based on the radiographic grading of RILI assessed by observer 1).

Variables	HR (95% CI)	P value
Age (per 1 year old)	0.95 (0.91–0.99)	0.01*
HOT (Yes vs No)	0.20 (0.04–0.88)	0.03*
Tumor diameter (per 1 mm)	1.05 (0.99–1.11)	0.09
Interstitial shadow (Yes vs No)	4.06 (1.13–14.5)	0.03*

Abbreviations: RILI, radiation-induced lung injury; HOT, home oxygen therapy.

*Statistically significant (p < 0.05).

## Discussion

This study aimed to assess the early and late CT appearance and severity of RILI after SBRT using a radiographic severity grading scale without consideration of the clinical presentation and treatment content for RILI. This attempt was considered to be successful because the results of each agreement were good. Although the agreement between the CTCAE grade and radiographic grading was not good, the result of MVA for the CT-based grade was interesting. The better the CT-based severity grading scale, the more information it can provide.

MVA for the CT-based grade assessed by observer 1 showed that age, HOT and interstitial shadow were significant predicting factors for grade 2 or more CT-based RILI. Older age was reported to be a risk factor for RILI [20–21]. In chemoradiotherapy for lung cancer, both the carboplatin/paclitaxel regimen and an age greater than 65 years were classified as high risks for RILI [22]. However, the result of this study showed the opposite: older age reduced HR, which suggested that the poorer tolerance to RILI in older age comes from age-related problems, such as comorbidities and frailty. In addition, HOT was a significant factor: patients treated with HOT had a lower CT-based RILI grade. Patients who received HOT have been thought to be susceptible to developing dyspnea and sometimes require an increased oxygen flow, but these points have not been well-studied. Our result suggested that older age and HOT patients have poorer tolerance to RILI, but this does not mean that older age and HOT patients have higher radiosensitivity. On the other hand, the presence of an interstitial shadow indicated a higher CT-based RILI grade, comparable to previous CTCAE-based findings [23]. RILI is induced not only by the progression of underlying disease but also by increased radiosensitivity, which sometimes lead to acute exacerbation of the underlying disease [24].

There have been some previous reports on RILI using CT appearance. Avanzo et al. regarded acute RILI as diffuse consolidation and a patchy consolidation and GGO as severe RILI. They reported V_5 Gy_, V_20 Gy_, the mean lung dose, and the number of fractions significantly correlated with severe RILI; the dose of the 50% probability of severe RILI was 73.0 Gy in 5 and 8 fractions [25–26]. Bernchou et al. divided CT appearance of acute RILI after conventional fractionated radiotherapy into 3 categories: interstitial changes, GGO, or consolidation [27]. Affecting factors all categories were that intervals between commencement of radiotherapy and follow-up CT scan and lung dose metrics. On the other hand, dosimetric factors such as V_5 Gy_ and V_20 Gy_ were not significant factors in this study because of the difference between “acute” and “late”. In regard to the severity of RILI, previous reports used an acute CT appearance; however, the late CT severity grading scale was used in current study. Dosimetric factors may be better predictors for an early CT appearance than a late CT appearance.

Although the agreement between the two observers was good, it fell short of excellent. A more precise definition would lead to better agreement. The difference between the training of the radiologist and the radiation oncologist may have also contributed to the not excellent agreement. One of the reasons will come from the difference between “patchy” and “diffuse”. This difference is defined as incompletely and completely filling the “high-dose region” in Linda’s criteria, the interpretation of which may differ between interobservers [17]. Dahele et al. defined the difference using an objective cutoff value that was more than 5 cm in the largest dimension or not [28]. This definition may offer better agreement between interobservers, but may offer a stronger effect of PTV on the radiological assessment of SBRT.

The date of the late CT appearance diagnosis also showed some difference. The intervals between SBRT and early CT diagnosis of observer 1 and observer 2 were almost the same and the averages were 4.4 months and 4.8 months, respectively. By contrast, intervals of late CT diagnoses of observer 1 and observer 2 had some differences: the averages were 22.2 months and 16.7 months, respectively. The interval periods were consistent with previous findings [17]. However, some difference of the intervals of a late CT diagnosis between observers indicated that prolonged or transitional shadows of an early CT appearance may have confused the observers. Defining this point more precisely may contribute to better agreement between observers.

There were several limitations in the current study. This study was a retrospective study conducted at a single institute with a limited sample size. The timing of follow-up CT was not constant. The number of patients receiving HOT and number of patients with interstitial shadows were small. Some possible factors, such as peripheral oxygen saturation, spirometry data and the serum KL-6 level, were lacking. A prospective study with a larger sample size is needed to overcome these limitations.

## Conclusions

In conclusion, the CT based appearance and severity of RILI were assessed with good agreement. Older age, receiving HOT and absence of an interstitial shadow were related to a lower grade of RILI. This relatively objective assessment could provide further information that has been masked by clinical presentation.

## Supporting information

S1 Data Relevant data.xls to this manuscript. (XLS)
